# Quercetagetin alleviates liver fibrosis in non-alcoholic fatty liver disease by promoting ferroptosis of hepatic stellate cells through GPX4 ubiquitination

**DOI:** 10.1186/s13020-025-01109-x

**Published:** 2025-06-16

**Authors:** Yuping Qiu, Shupei Li, Mingzuo Jiang, Ang Huang, Ya Yang, Xi Chen, Hui Li, Zhizhou Yang, Juan Wei, Ji Xuan

**Affiliations:** 1 Department of Gastroenterology, School of Medicine, Jinling Hospital, Nanjing University, 305 Zhongshan East Road, Nanjing, 210002 China; 2 Jinling Clinical Medical College, Nanjing University of Chinese Medicine, Nanjing, China; 3 Department of Gastroenterology and Hepatology, The First Medical Center of PLA General Hospital, Beijing, China; 4 Department of Gastroenterology, Affiliated Hospital of Integrated Traditional Chinese and Western Medicine, Nanjing University of Chinese Medicine, 100 Shizi Street, Nanjing, 210028 China; 5 Department of Outpatient and Emergency, Qinhuai Medical District, Affiliated Hospital of Medical School, Jinling Hospital, Nanjing University, 305 Zhongshan East Road, Nanjing, 210002 China; 6 Department of Emergency Medicine, The First School of Clinical Medicine, Southern Medical University, Guangzhou, China; 7 Department of Gastroenterology, Jinling Hospital, The First School of Clinical Medicine, Southern Medical University, Guangzhou, China; 8 Department of Gastroenterology, The First School of Clinical Medicine, Southern Medical University, Guangzhou, China

**Keywords:** Non-alcoholic fatty liver disease, Hepatic stellate cells, Quercetagetin, Ferroptosis, GPX4 ubiquitination

## Abstract

**Background:**

Lang Qing A Ta (Huagan Tongluo Fang, HGTLF) is a Tibetan medicine with significant anti-liver fibrosis effects and good efficacy in the treatment of liver diseases, including non-alcoholic fatty liver disease (NAFLD). Quercetagetin (QG) has been identified as an active ingredient of HGTLF that is absorbed into the blood. This study aims to investigate the role of QG in the anti-liver fibrosis effect of HGTLF in NAFLD.

**Methods:**

CCl_4_ injection-induced liver fibrosis and high-fat, high-cholesterol diet-induced non-alcoholic steatohepatitis (NASH) mouse models were established. Transforming growth factor-β1 (TGF-β1)-induced hepatic stellate cells (HSCs) were used as in vitro models. The effect of QG on the stability and degradation pathway of glu-tathione peroxidase 4 (GPX4) protein was investigated.

**Results:**

QG improved liver function and hyperlipidemia in CCl_4_-injected mice and NASH mice, and alleviated hepatic lipid deposition and hepatic fibrosis. TGF-β1 treatment promoted the expression of α-smooth muscle actin and fibrosis-related genes, while QG reversed this phenomenon and inhibited HSC activation. QG increased the intracellular labile iron pool and lipid reactive oxygen species in HSCs. Treatment with the ferroptosis inhibitor ferrostatin-1 reversed the inhibitory effect of QG on TGF-β1-induced HSC activation. QG reduced GPX4 protein stability and regulated GPX4 K167 ubiquitination via the membrane-associated ring-CH-type finger 8 (MARCHF8)-mediated ubiquitin–proteasome pathway. Interference with MARCHF8 attenuated the effect of QG and promoted HSC activation induced by TGF-β1.

**Conclusion:**

QG, the active ingredient of HGTLF, can induce ferroptosis of HSCs by targeting the degradation of GPX4 through ubiquitination and inhibit HSC activation, thereby alleviating liver fibrosis in NAFLD.

**Supplementary Information:**

The online version contains supplementary material available at 10.1186/s13020-025-01109-x.

## Introduction

Non-alcoholic fatty liver disease (NAFLD) refers to a clinicopathological syndrome which is caused by other factors other than alcohol and definite liver damage, including simple hepatic steatohepatitis, non-alcoholic steatohepatitis (NASH), and hepatocellular carcinoma associated with NASH [1]. Currently, there is no specific drug to treat NAFLD, and lifestyle modification through dietary changes, weight control, and increased physical activity remains the cornerstone of NAFLD management, but most people have difficulty adhering to it for long periods of time [2]. Therefore, it is of great importance to explore new methods and molecular targets for the prevention and treatment of NAFLD.

Tibetan medical system is the treasure of traditional Chinese medicine, with a complete theoretical system and a long history and culture. A growing number of studies have found that Tibetan medicine can prevent and treat NAFLD, including triphala [3], sea buckthorn [4], gentiana scabra [5], etc. Lang Qing A Ta, also known as Huagan Tongluo Fang (HGTLF), is a famous empirical formula for liver treatment in Tibetan medicine. It is composed of artificial bezoar, saffron, fecal trogopterori, astragalus, macelignan, agarwood, etc., has the effect of heat-clearing and detoxifying, soothing liver and bile galling. Based on the theory of stasis and heat, this formula has achieved fruitful results in the treatment of liver fibrosis, which provides a new idea for the clinical application of Tibetan medicine. Our research group has previously found that HGTLF has relatively significant anti-fibrosis ability in CCl_4_-induced liver fibrosis mouse model, and confirmed the role of crocin, an important component of HGTLF, in inhibiting HSC activation and ameliorating liver fibrosis [6, 7]. In addition, our research group has completed the pharmacological and toxicological evaluation of HGTLF and tested it in clinical treatment, and found that HGTLF can significantly improve liver function and the degree of fatty liver in patients with liver fibrosis caused by NAFLD. However, the mechanism of HGTLF in anti-liver fibrosis of NAFLD remains unclear.

Hepatic stellate cells (HSCs) play an important role in the onset and development of liver fibrosis in NAFLD [8]. Under physiological conditions, HSCs are quiescent. However, the onset of inflammation and oxidative stress caused by repeated insults such as chronic viral infection, alcohol addiction, and NASH can transform quiescent HSCs into activated HSCs. Activated HSCs transform into myofibroblasts that produce large amounts of collagen and express α-smooth muscle actin (α-SMA), leading to progressive accumulation of extracellular matrix (ECM) [9]. Ferroptosis, as a form of cell death, plays an important role in the regulation of various pathological processes, including liver fibrosis. The mechanism of ferroptosis is complex and involves the iron replacement pathway, the glucathione peroxidase 4 (GPX4)-dependent signaling pathway, and the lipid peroxidation chain reaction [10, 11]. It’s worth noting that the induction of HSC ferroptosis can effectively reduce the excessive proliferation and deposition of ECM in liver tissue and ultimately delay the progression of liver fibrosis [12, 13]. However, the relationship between HSC activation and ferroptosis in NAFLD needs further investigation.

In this study, a metabolomics method of ultra-high performance liquid chromatography tandem mass spectrometry (UPLC-MS/MS) was developed for the detection of metabolites in blood, and high-performance liquid chromatography (HPLC) identified quercetagetin (QG), a major flavonoid, in liver tissues of high-fat, high-cholesterol diet (HFHCD) mice administered with HGTLF. Recent studies have confirmed that QG can alleviate zearalenone-induced liver injury in rabbits [14], but whether it has a protective effect against hepatitis caused by other factors is not clear. Therefore, this study aims to explore the molecular mechanism of QG in inhibiting HSC activation and playing an anti-liver fibrosis role in NAFLD transformation by using transforming growth factor-β1 (TGF-β1)-induced HSC model in vitro and CCl_4_ injection or HFHCD-treated liver fibrosis model in vivo.

## Materials and methods

### Establishment of CCL_4_-induced liver fibrosis model and HFHCD-induced NASH model

The 6-week-old SPF C57BL/6 J mice with a body weight of (20 ± 2) g were purchased from Changzhou Cavens Laboratory Animal Co. Ltd. The experimental program was approved by the Animal Ethics Committee of Jinling Hospital, Nanjing University, School of Medicine. After 1 week of adaptive feeding, 10% CCl_4_ corn oil (1 kg/mL, Shanghai Macklin Biochemical Technology Co., Ltd.) was injected intraperitoneally three times a week for 4 weeks to induce liver fibrosis in mice. Mice in the administration group were given low (2.75 mg/kg/d) or high (11 mg/kg/d) doses of QG by gavage daily for 4 weeks [15]. Mice in the normal group were intraperitoneally injected with the same volume of corn oil and/or gavaged with the same volume of normal saline at the same time point and then grouped into corn oil, corn oil + L-QG, corn oil + H-QG, CCl_4_, CCl_4_ + L-QG, CCl_4_ + H-QG groups (N = 5/group). To establish a NASH mouse model, 6-week-old C57BL/6 J mice were fed HFHCD for 16 weeks, while control mice were fed normal chow diet (NCD). Mice in the administration group were gavaged with high doses (11 mg/kg/d) of QG daily for 16 weeks and then grouped into NCD, NCD + QG, HFHCD, HFHCD + QG groups (N = 5/group) [16].

Twelve hours after the last administration, the mice were weighed and recorded, and 0.3% pentobarbital was injected intraperitoneally for anesthesia. Blood samples were collected from the eyeballs, centrifuged at 3000 rpm for 15 min at 4 ℃, and the top layer of serum was collected for detection of serum liver function indices. Mice in each group were sacrificed after blood collection, and liver tissues were weighed and recorded. The liver index (liver weight/body weight of mice × 100%) was calculated. The liver tissues were then used for triglyceride (TG) and total cholesterol (TC) content determination and pathological observation.

### Non-targeted metabolomics analysis and HPLC analysis

The chemical compounds of HFHCD oral solution were analyzed by UPLC-MS/MS. The Ultimate 3000 L C in series with a Q-Exactive (Thermo Fisher Scientific) was used to separate and detect secondary metabolites, and the chromatographic column used was the ACQUITY UPLC HSS T3 column 1.8 μm (2.1 × 100 mm). Mass spectrometry was performed using positive and negative electron spray ionization modes. The metabolites were purified by Suzhou Panomic Biomedical Technology Co.

Subsequently, mice were intragastrically administered HGTLF (11 g/kg) for 7 d after establishment of the HFHCD mouse model and grouped into HFHCD and HFHCD + HGTLF. Serum from the mice was collected for non-targeted metabolomics by UPLC-MS/MS. Serum samples were collected from the – 80 ℃ refrigerator and thawed on ice, and subsequent operations were performed on ice. Then, 50 mg of the sample was added to 2 mL centrifuge tubes, and 800 μL of 80% methanol (Thermo Fisher Scientific) was added to each centrifuge tube, then thoroughly mixed by shaking for 1 min. The mixture was centrifuged at 4 °C, 12000 rpm for 15 min, and the supernatant was transferred to a new centrifuge tube. Then, 200 μL of the supernatant was mixed with 5 μL of dichlorophenylalanine (Aladdin) and then filtered through a 0.22 μm membrane to obtain the sample for LC–MS analysis. The Ultimate 3000 LC combined with a Q Exactive (Thermo Fisher Scientific) was used for metabolite separation and detection. The chromatographic column used was the ACQUITY UPLC HSS T3 1.8 μm (2.1 × 100 mm) column. Mass spectrometry was performed in positive ion mode and negative ion mode, and then differential metabolites were identified between groups.

Liver tissues were collected from mice in the HFHCD and HFHCD + HGTLF groups and HPLC was performed to determine the concentration of all flavonoids. Chromatographic separation was performed on an Agilent TC-C18 column (4.6 mm × 250 mm, 5 μm, Agilent). The detection wavelength was 360 nm.

### Detection of liver function

After blood sampling from the abdominal aorta, arterial blood was allowed to stand for 1 h, centrifuged at 3000 rpm, 4 ℃ for 15 min, and the supernatant was removed. An automated chemistry analyzer (AU5800, BECKMAN COULTER) was used to determine serum aspartate aminotransferase (AST), alanine aminotransferase (ALT), alkaline phosphatase (ALP), TG, and TC levels.

Then, an appropriate amount of liver tissue from mice in each group was added to RIPA lysate (Beyotime) for grinding and homogenization. After centrifugation at 12,000 rpm for 30 min (repeated 2–3 times), the supernatant was combined, and then the contents of TG and TC in liver tissues were detected by microplate reader according to the corresponding kit instructions (Nanjing Jiancheng Bioengineering Institute).

### Pathological changes of liver tissues

The same portion of the left lateral lobe of the liver from mice in each group was fixed in 4% paraformaldehyde solution (Sigma) for 24 h, embedded in paraffin, and sectioned (4 μm). Hematoxylin–eosin (HE) staining kit (Beijing Solarbio Science & Technology Co., Ltd.) and oil red O staining kit (Wuhan Servicebio Technology Co., Ltd.) were used to observe the pathological structure of liver tissues. Masson's staining kit (Beijing Solarbio Science & Technology Co., Ltd.) and Sirus Red staining kit (Beijing Solarbio Science & Technology Co., Ltd.) were used to observe fibrosis changes of liver tissues.

### Determination of total collagen content

Total collagen content in mouse lung tissues was analyzed by the hydroxyproline (HYP) method for quantitative assessment of lung tissue fibrosis. According to the kit instructions (K218, Biovision), lung tissues were extracted from each group for grinding and homogenization. After the addition of 6 mol/L hydrochloric acid, the tissues were transferred to 1.5 mL EP tube, boiled in a water bath, and then centrifuged at 12000 rpm at 25 ℃ for 25 min. Finally, NaOH was used to adjust the pH to 6–8. The OD value was determined at 560 nm with the supernatant, and the content of HYP was calculated.

### Immunohistochemical staining

HSC activation markers α-SMA and desmin were detected by immunohistochemical staining. Paraffin sections were heated, deparaffinized in xylene, hydrated in conventional graded alcohol, rehydrated in citric acid antigen, blocked with peroxidase and goat serum, and incubated overnight with primary antibodies (mouse anti-desmin, 1:100, BOSTER; rabbit anti-α-SMA, 1:100, BOSTER). The sections were washed three times with PBS solution, incubated with secondary antibodies for 30 min, stained with 3,3 N-diaminobenzidine tetrahydrochloride (DAB, Beyotime), counterstained with hematoxylin, routinely dehydrated and transparentized, and sealed with neutral rubber before microscopic examination (Olympus).

### Isolation of primary mouse HSCs and hepatocytes (Hpys)

Mice were anesthetized with pentobarbital sodium, and the liver and postcava were exposed. An indwelling needle was inserted into the postcava, and Hank's balanced salt solution (Thermo Fisher Scientific) and 0.01% IV collagenase (Sigma) were perfused sequentially in situ. At the end of perfusion, the cell suspension was filtered through a 70 μm cell strainer to collect Hpys. Primary mouse HSCs were then collected by Percoll density gradient centrifugation. Hpys and HSCs were cultured in Dulbecco's modified Eagle's medium (DMEM) complete medium (HyClone) containing 10% fetal bovine serum and 1% penicillin–streptomycin at 37 °C and 5% CO_2_.

### Cell treatment

Human HSCs (LX-2 cells) were purchased from Procell Life Science & Technology Co., Ltd. and cultured in DMEM medium containing 10% FBS and 1% penicillin–streptomycin at 37 ℃ and 5% CO_2_. Primary mouse HSCs and LX-2 cells were treated with 5 ng/mL TGF-β1 (PeproTech) for 24 h to induce activation. In the TGF-β1 + QG group, cells were simultaneously treated with low (10 μM), medium (30 μM), and high (50 μM) concentrations of QG and TGF-β1 for 24 h. In the QG + ferrostatin-1 (Fer-1)/zVAD-FMK (zVAD)/necrostatin-1 (Nec-1) group, cells were simultaneously treated with QG and 1 μM Fer-1, 50 μM zVAD, or 10 μM Nec-1 for 24 h. In the TGF-β1 + QG + Fer-1 group, cells were simultaneously treated with TGF-β1, QG, and 1 μM Fer-1 for 24 h. When cell confluence was 80%, plasmids (OE-MARCHF3, OE-MARCHF8, OE-MARCHF11, negative control OE-NC, shMARCHF8, or negative control shNC) were transfected into primary mouse HSCs using the Lipofectanine3000 transfection kit (Invitrogen). QG treatment was performed 24 h after transfection.

### Immunofluorescence staining

The harvested cells were rinsed with PBS and fixed with 40 g/L paraformaldehyde solution. After rinsing with PBS, the cells were treated with 0.5% Triton X-100 solution and 5% goat serum sealant solution (Beijing Solarbio Technology Co. Ltd.). Then, α-SMA antibody (ab5694, abcam) was added and incubated at 4℃ for 12 h. After the cells were completely washed with PBS, they were incubated with secondary antibody (ab150077, abcam) at room temperature for about 1 h. The cell nucleus was re-stained with DAPI solution (2 μg/mL, Sigma) at room temperature for 15 min, and the film was observed and photographed under a fluorescence microscope (Olympus) after sealing.

### FerroOrange staining

HSCs were seeded in a confocal or glass-bottomed culture dish at 37 °C with 5% CO_2_ overnight. The culture medium was aspirated and the cells were washed three times with serum-free medium or HBSS to remove any extracellular iron ions. A 1 µM FerroOrange working solution was then prepared by diluting a 1 mM stock solution of FerroOrange in HBSS. The FerroOrange working solution was added to the cells and incubated for 30 min at 37 °C in the dark. After incubation, the cells were rinsed twice with HBSS or serum-free medium to remove unbound dye. Cells were observed by confocal fluorescence microscopy. FerroOrange has an excitation maximum at 543 nm and an emission maximum at 580 nm. Fluorescence intensity increases in the presence of intracellular Fe^2+^, indicating iron overload.

### Quantitative reverse transcription-polymerase chain reaction (qRT-PCR)

The mRNA levels of α-2 smooth muscle actin (ACTA2), laminin 1 (LAMA1), collagen type 1 α-1 (COLlA1), fibronectin 1 (FN1), and GPX4 were detected by qRT-PCR. After cells were lysed in Trizol solution (Invitrogen), total RNA was extracted by the chloroform-isopropyl alcohol method, and the purity and concentration of the extracted RNA were determined using a micronucleic acid analyzer (Nanodrop2000). cDNA was synthesized by reverse transcription according to the PrimeScript™ RT Master Mix Kit (TaKaRa). PCR amplification was performed using SYBR Premix Ex Taq™ II kit (TaKaRa) with cDNA as template. GAPDH was used as an internal parameter in the experiment. Relative expression was calculated using the 2^−ΔΔCt^ method. The following primers were used in this study: mGAPDH sense: 5′-TGTTGAAGTCGCAGGAGACAACCT-3′, antisense: 5′-AACCTGCCAAGTATGATGACATCA-3′; mACTA2 sense: 5′-CAGGGAGTAATGGTTGGAAT-3′, antisense: 5′-TCTCAAACATAATCTGGGTCA-3′; mCol1a1 sense: 5′-CATGAGCCGAAGCTAACCC-3′, antisense: 5′-TGTGGCAGATACAGATCAAGC-3′; mLAMA1 sense: 5′-CAACTGCTCGCAGAATACCA-3’, antisense: 5′-GCCACTTTCCATTGGCTAAA-3′; mFN1 sense: 5′-GAAGGTTTGCAACCCACTGT-3′, antisense: 5′-CATCCTCAGGGCTCGAGTAG-3′; mGPX4 sense: 5′-CTCCGAGTTCCTGGGCTTGTG-3′, antisense: 5′-CCGTCGATGTCCTTGGCTGAG-3′; hGAPDH sense: 5′-ACAACTTTGGTATCGTGGAAGG-3′, antisense: 5′-GCCATCACGCCACAGTTTC-3′; hLAMA1 sense: 5′-GCAGCTCTGGAGTACGTTCC-3′, antisense: 5′-CCTTTGCCAAGGTCAAACAT-3′; hACTA2 sense: 5′-AAAAGACAGCTACGTGGGTGA-3’, antisense: 5′-GCCATGTTCTATCGGGTACTT-3′; hCol1a1 sense: 5′-:GAGGGCCAAGACGAAGACATC-3′, antisense: 5′-CAGATCACGTCATCGCACAAC-3′; hFN1 sense: 5′-CGGTGGCTGTCAGTCAAAG-3′, antisense: 5′-AAACCTCGGCTTCCTCCATAA-3’.

### Lactate dehydrogenase (LDH) release assay

Cells in different treatment groups (5 × 10^3^ cells/well) were seeded into 96-well plates and cultured for 48 h, and the cell supernatant was collected. After centrifugation at 1900 rpm for 5 min at room temperature, the supernatant was transferred to a new 96-well plate, and 60 μL LDH assay solution (Beyotime) was added to each well. After incubation for 30 min at room temperature, the OD value was measured at 490 nm.

### Flow cytometry analysis

After trypsin digestion, the cells were washed twice with PBS and incubated with 0.2 μmol/L calcien acetoxymethyl ester [C-AM, Sangon Biotech (Shanghai) Co., Ltd]. After three washes with PBS, the cells were treated/not treated with 100 μmol/L deferiprone (MedChemExpress) for 1 h at 37 °C. The mean fluorescence intensity (MFI) of C-AM was detected by a flow cytometer (BD Biosciences) to represent the intracellular labile iron pool (LIP).

Cells were incubated with BODIPY 581/591 C11 (C11-Bodipy) dye (Thermo Fisher Scientific) at 37 °C for 20 min. After three washes with PBS, cells were resuspended in 500 μL PBS, and lipid reactive oxygen species (ROS) were detected by flow cytometer (BD Biosciences).

### Western blot assay

The protein expressions of transferrin receptor (TFRC), solute carrier family 40 member 1 (SLC40A1), solute carrier family 7 member 11 (SLC7A11), solute carrier family 3 member 2 (SLC3A2), nuclear receptor co-activator 4 (NCOA4), GPX4, ferroptosis suppressor protein 1 (FSP1), membrane-associated ring-CH-type finger 1 (MARCHF1), MARCHF3, MARCHF8, and MARCHF11 were detected by Western blot assay. Total protein was extracted from each group of cell lysates. After protein concentration was determined by BCA kit (Beyotime), the same amount of protein was added to sample buffer and heated for SDS-PAGE electrophoresis. The separated protein was transferred to PVDF membrane by wet transfer method, sealed in skim milk for 2 h, then primary antibodies were added and incubated at 4℃ overnight. After washing with Tris buffer saline Tween-20, cells were incubated with secondary antibody and incubated at room temperature for 2 h. Finally, the color was developed with ECL kit (Biosharp) and photographed with a gel imager.

### Protein stability test and treatment with proteasome inhibitor MG132

The cycloheximide (CHX) chase assay was used to evaluate the stability of GPX4 protein. After cell digestion, 50 μg/mL CHX (MedChemExpress) was added to treat cells, and cells were harvested at 0, 3, 6, and 9 h for Western blot analysis. MG132 was used to demonstrate whether GPX4 is degraded by the ubiquitin–proteasome system. 10 µM MG132 (Sigma) or 25 µM chloroquine (CQ, MedChemExpress) was added and cultured for 4 h, and cells were harvested for Western blot analysis.

### Ubiquitination detection

The ubiquitination experiment was performed as described previously [17]. Briefly, primary HSCs in the QG-treated group or MARCHF8 overexpression group were added to 10 µM MG132 for 8 h. Cells were collected and centrifuged at 4 ℃, 3000 rpm for 5 min, then the supernatant was discarded and placed on ice. Then, 400 μL N-(2-hydroxyethyl) piperazine-N'-2-ethanesulfonic acid (HEPES, MedChemExpress) lysate was added for resuspension, and after ultrasonic lysis, cells were centrifuged at 4 ℃, 12000 rpm for 10 min. The 40 μL supernatant was added to 2 × loading buffer and immersed in water at 100 ℃ for 5 min to detect the expression of intracellular proteins. The remaining 360 μL supernatant was added with 2 μL IgG antibody and incubated at 4 ℃ for 1 h. Then, 40–60 μL protein A/G PLUS agarose (Bimake) was added and incubated at 4 ℃ for 2 h. The magnetic beads were removed, and the supernatant was mixed with antibody and incubated at 4 ℃ for 3 h. Then, 40–60 μL Protein A/G PLUS Agarose was added and incubated at 4 ℃ overnight. After centrifugation at 3000 rpm for 3 min at 4 ℃, 960 μL supernatant was discarded, and the remaining 40 μL was added to 2 × loading buffer, mixed well, and then bathed in water at 100 ℃ for 10 min. The level of GPX4 ubiquitination was detected by Western blot assay. To investigate the specific site of GPX4 ubiquitination, 293 T cells (Procell Life Science & Technology Co., Ltd.) were transfected with Flag-MARCHF8 plasmid and Myc-GPX4 (WT), Myc-GPX4 (K161R), Myc-GPX4 (K167R), respectively.

### Co-immunoprecipitation (Co-IP) assay

After 30 min of lysis, the cells were centrifuged at 13000 rpm for 15 min at 4 ℃. Then, 1/10 of the supernatant was retained as input and the rest was mixed with GPX4 or MARCHF8 antibody and corresponding control IgG antibody. After incubation at 4 ℃ for 3 h, an appropriate amount of protein A/G magnetic beads was added and incubated overnight at 4 ℃. The next day, the magnetic beads were washed 4 times with cold lysis buffer for 10 min each time. After the washing solution was discarded, 2 × loading buffer was added and heated in a 98℃-water bath for 10 min. The supernatant was collected, and the protein was detected by Western blotting.

### Statistical analysis

SPSS 25.0 statistical software was used for statistical analysis, and data were expressed as mean ± SD. Unpaired t-test was used to compare results between two groups. One-way ANOVA with Bonferroni correction was used to compare results between multiple groups. *P* < 0.05 was considered statistically significant.

## Results

### QG alleviates liver fibrosis in CCl_4_-injected mice

The study of drug pharmacokinetics is a hot spot in traditional pharmaceutical research. Metabolomic analysis of the differential changes of metabolites in vivo after drug administration is helpful for drug target discovery and elucidation of disease pathogenesis. The chemical components of HGTLF and the components that migrated into the blood were determined by UPLC-MS/MS (Fig. S1A-B). A total of 1518 components were identified in HGTLF (Supplementary Table), among which flavonoids were the most abundant (159, 30.4%), followed by terpenoids (92, 17.59%) and phenylpropanoids (57, 10.9%) (Fig. S1C). Drug-containing serum (HFHCD + HGTLF group) and control serum (HFHCD group) were subjected to non-target metabolomic sequencing. The metabolites obtained from the sequencing of the two groups were intersected to obtain the common metabolites. All common metabolites in the drug-containing serum group were removed, and the remaining metabolites were the unique metabolites in the drug-containing serum group, with a total of 260 (Supplementary Table). In addition, 260 unique metabolites of the drug-containing serum group were compared with 1518 secondary metabolites of HGTLF, and 106 blood components of HGTLF were determined (Fig. S1D). HPLC technology was also used to determine the concentration of all flavonoids (fustin, isoformononetin, liquiritigenin, marini, patuletin, quercetagetin, senegalensin) in liver tissue after HGTLF administration. The results showed that the concentration of QG was highest in mouse liver tissue (Fig. S1E).

**Fig. 1:**
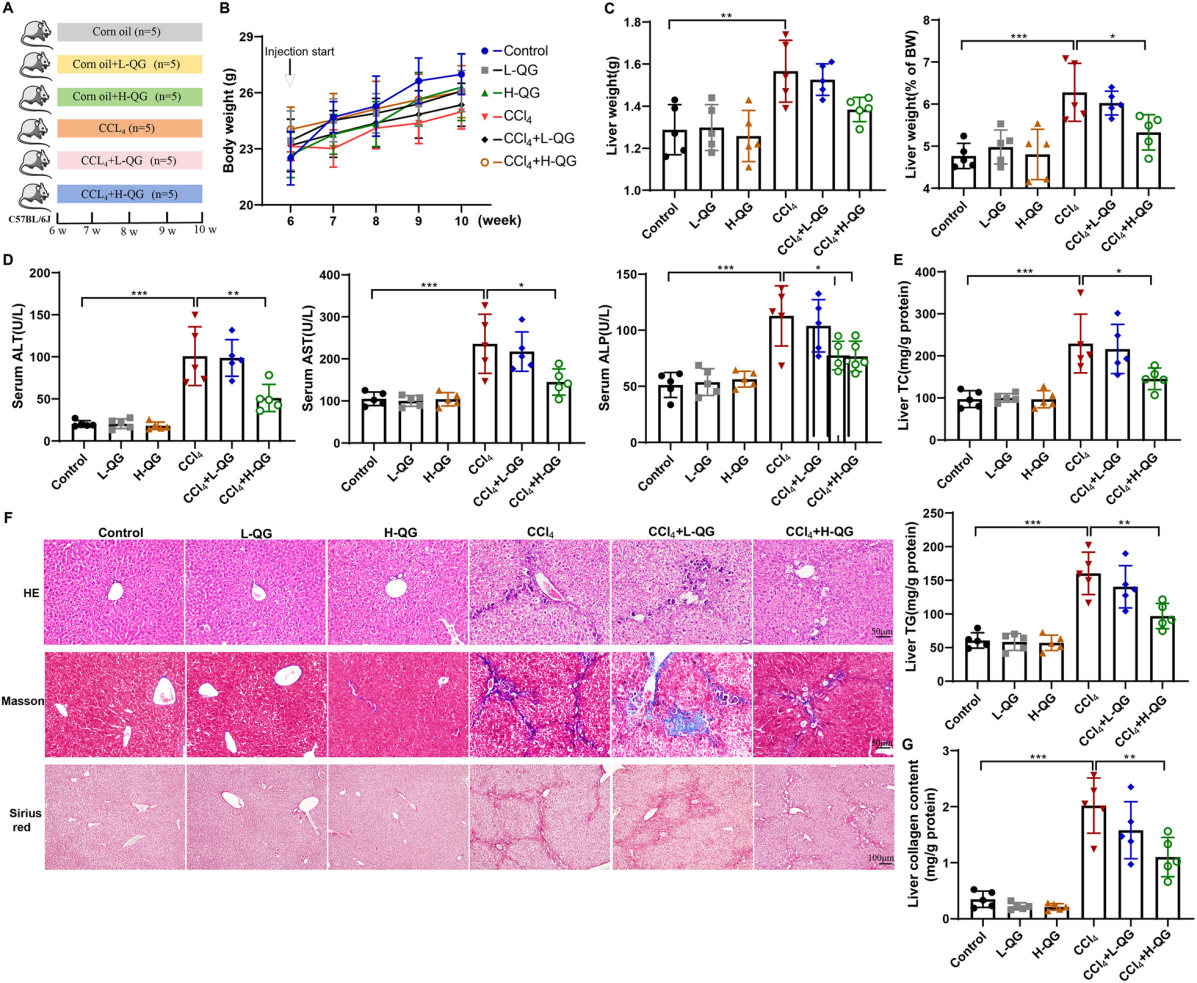
Effects of QG on body weight, liver function and liver fibrosis in CCl_4_-injected mice. **A** Mice were divided into control (corn oil), corn oil + L-QG, corn oil + H-QG, CCl_4_, CCl_4_ + L-QG, and CCl_4_ + H-QG groups (N = 5/group). **B–C** Body weight and liver weight of mice in each group were determined, and liver index was calculated. **D** Blood samples were collected from mice in each group for the detection of AST, ALT and ALP levels. **E** Liver tissues were collected from mice in each group for the determination of TC and TG contents. **F** Liver tissues from mice in each group were stained with HE (scale bar, 50 μm), Masson (scale bar, 50 μm), and Sirius red (scale bar, 100 μm). **G** HYO levels were detected in the liver tissues of mice in each group to characterize collagen content. **P < 0.01, ***P < 0.001 *vs* control; *P < 0.05, **P < 0.01 *vs* CCl_4_

The hepatotoxic substance CCl_4_ is one of the most effective ways to damage hepatocytes by exogenous toxins, and the CCl4-induced liver fibrosis model is also one of the classic animal models [18]. To verify the effects of QG on body weight, liver function and liver fibrosis in vivo, mice were divided into control, corn oil + L-QG, corn oil + H-QG, CCl_4_, CCl_4_ + L-QG, CCl_4_ + H-QG groups (Fig. 1A). CCl_4_ treatment had no effect on the body weight of mice, and QG treatment had no effect on the body weight of CCl_4_ model mice (Fig. 1B). Figure 1C showed that CCl_4_ injection significantly increased liver weight and liver index compared with the control group. Compared with the CCl_4_ group, L-QG or H-QG had no significant effect on the liver weight of mice, whereas H-QG decreased the liver index. Next, the liver function of mice in each group was determined. Compared with the control group, CCl_4_ injection increased serum AST, ALT, ALP levels and liver TC, TG contents, indicating that the liver function of mice was impaired and lipid deposition occurred in the liver, which could be alleviated by H-QG (Fig. 1D–E). HE staining showed that the structure of liver lobules was intact and the morphology of hepatocytes was normal in the normal group, the structure of liver lobules was damaged and the arrangement of hepatocytes was disordered in the CCl_4_ and CCl_4_ + L-QG group, and the structure of liver lobules was generally normal and the arrangement of hepatocytes was basically ordered in the H-QG treatment group (Fig. 1F). Masson's staining and Sirius red staining showed that the liver of the normal group contained a small amount of collagen fibers, and the liver collagen fibers of the CCl_4_ and CCl_4_ + L-QG groups proliferated significantly, and the proliferation of collagen fibers was reduced after H-QG treatment (Fig. 1F). In addition, the HYP method was used to measure the relative content of collagen in the liver of mice, and the results showed that H-QG reduced the content of collagen in the liver of mice injected with CCl_4_ (Fig. 1G). These results indicated that QG at a high dose exerted significant inhibitory effects on body weight, liver function, and liver fibrosis in CCl_4_-injected mice.

### QG alleviates hepatic lipid deposition and fibrosis in NASH model mice

**Fig. 2:**
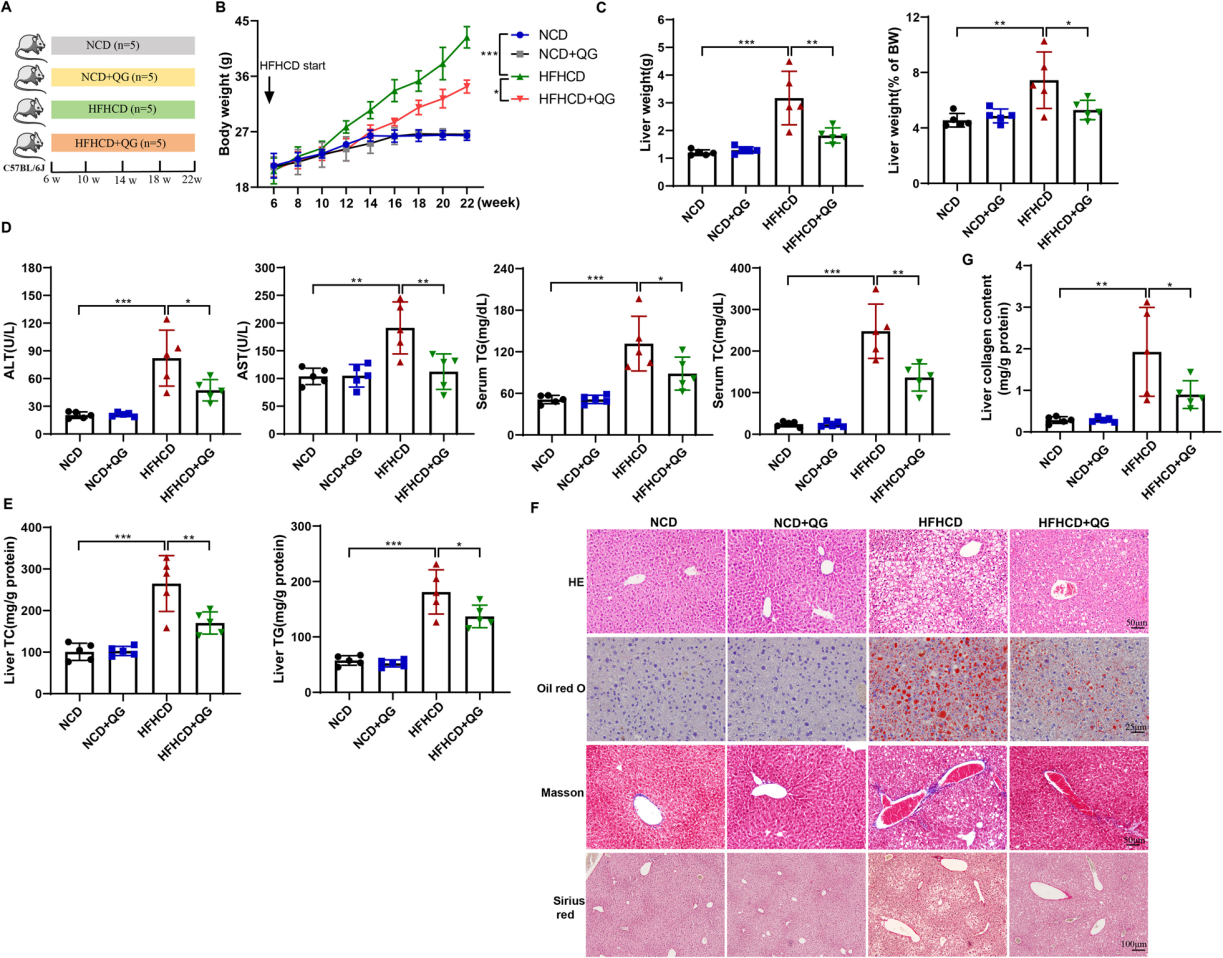
Effect of QG on hepatic lipid deposition and fibrosis in NASH model mice. **A** Mice were divided into NCD, NCD + QG, HFHCD, and HFHCD + QG groups (N = 5/group). **B–C** Body weight and liver weight of mice in each group were recorded, and liver index was calculated. **D** Blood samples of mice in each group were collected for the detection of AST, ALT, TC and TG levels. **E** The liver tissues of mice in each group were collected for the detection of TC and TG levels. **F** Liver tissues from mice in each group were stained with HE (scale bar, 50 μm), oil red O stain (scale bar, 25 μm), Masson's stain (scale bar, 50 μm), and Sirius red (scale bar, 100 μm). **G** HYO levels were detected in the liver tissues of mice in each group to characterize collagen content. **P < 0.01, ***P < 0.001 *vs* NCD; *P < 0.05, **P < 0.01 *vs* HFHCD

Next, mice were treated with high-dose QG and divided into NCD, NCD + QG, HFHCD, HFHCD + QG groups (Fig. 2A). QG could inhibit HFHCD-induced increases in body weight, liver weight, and liver index of mice (Fig. 2B–C). QG administration reduced the levels of serum AST, ALT, TC, and TG and the levels of liver serum TC and TG in HFHCD mice, indicating that QG administration had an ameliorative effect on liver function and hyperlipidemia in HFHCD mice (Fig. 2D–E). Compared with NCD and NCD + QG groups, the structure of liver lobules was severely damaged, fat vacuoles appeared, many red lipid droplets accumulated, and obvious fibrosis occurred in HFHCD group, while QG could alleviate the above pathologies (Fig. 2F). In addition, QG administration reduced the collagen content in the liver of HFHCD mice (Fig. 2G). We also examined the effect of QG on the activation of lipid metabolism-related molecules (AMPK, SREBP-1c, and PPAR-γ) in the livers of HFHCD mice, and the results showed that QG promoted AMPK activation while inhibiting SREBP-1c and PPAR-γ expression (Fig. S2A), suggesting that QG ameliorates diet-induced lipid deposition by regulating liver lipid metabolism. In the in vitro experiments, we used a free fatty acid (FFA)-induced lipid accumulation model in mouse hepatocytes [19]. The results showed that QG had no significant effect on FFA-induced inhibition of AMPK activation and increased SREBP-1c and PPAR-γ expression (Fig. S2B). Therefore, we suggest that QG alleviates liver steatosis by inhibiting HSC activation and thereby regulating lipid metabolism-related signaling pathways such as AMPK. Taken together, the above results indicated that QG administration in vivo could ameliorate liver steatosis and fibrosis in the HFHCD-induced mouse model.

### QG inhibits TGF-β1-induced HSC activation and promotes HSC death in vitro

**Fig. 3:**
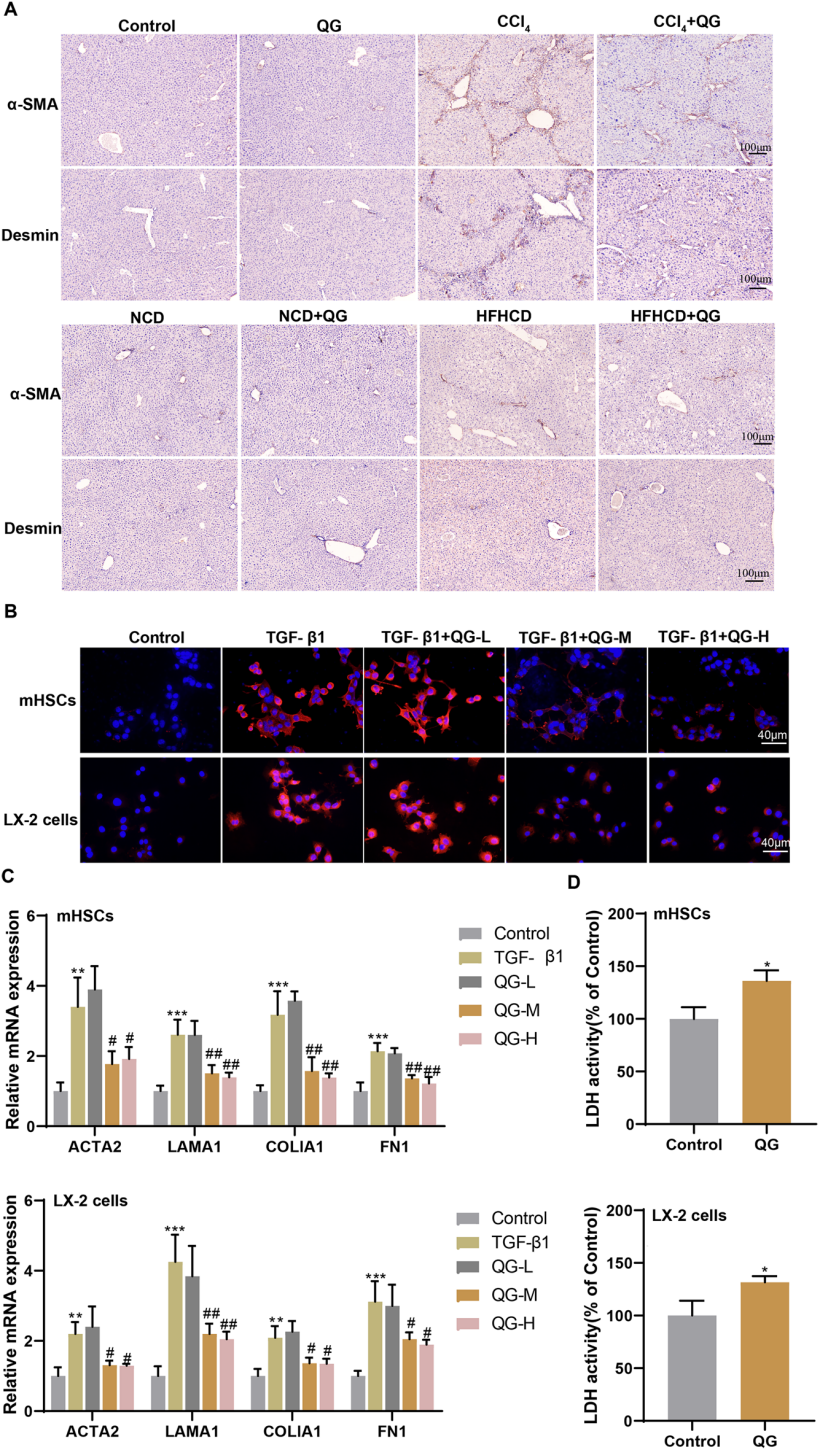
QG inhibits TGF-β1-induced HSC activation and promotes HSC death in vitro. **A** The positive expressions of α-SMA and desmin in liver tissues of control, QG, CCl_4_, CCl_4_ + QG group mice or NCD, NCD + QG, HFHCD, HFHCD + QG group mice were detected by immunohistochemical staining (scale bar, 100 μm). **B–C** Primary mouse HSCs or LX-2 cells were divided into control, TGF-β1, TGF-β1 + QG-L, TGF-β1 + QG-M, and TGF-β1 + QG-H groups. **B** Positive expression of α-SMA was detected by immunofluorescence staining (scale bar, 40 μm). **C** The mRNA levels of ACTA2, LAMA1, COLlA1, and FN1 were detected by qRT-PCR. *P < 0.05, **P < 0.01, ***P < 0.001 *vs* control; ^#^*P* < 0.05, ^##^*P* < 0.01 *vs* TGF-β1. **D** Primary mouse HSCs or LX-2 cells were divided into control and QG groups. The LDH activity was detected. *P < 0.05 *vs* control

Activated HSCs play a key role in the initiation and development of liver fibrosis and express markers such as α-SMA and desmin. The results of immunohistochemical staining showed that QG reduced the number of α-SMA- and desmin-positive cells in the liver tissues of CCl_4_- and HFHCD-induced liver fibrosis mice (Fig. 3A), suggesting that QG could inhibit the activation of HSCs. Next, mouse primary HSCs or LX-2 cells were divided into control, TGF-β1, TGF-β1 + QG-L, TGF-β1 + QG-M, and TGF-β1 + QG-H groups. Figure 3B showed that TGF-β1 treatment promoted α-SMA-positive expression, but both QG-M and QG-H inhibited α-SMA-positive expression, indicating that QG could inhibit TGF-β1-induced activation of HSCs. qRT-PCR results showed that QG-M and QG-H inhibited the increase in mRNA levels of ACTA2, LAMA1, COLlA1, and FN1 induced by TGF-β1 (Fig. 3C). The LDH results showed that QG treatment could significantly increase the LDH activity, suggesting that the death of HSCs was promoted (Fig. 3D). Based on the above results, we concluded that QG could inhibit TGF-β1-induced activation of HSCs and might be associated with cell death.

### QG promotes ferroptosis of HSCs and inhibits HSC activation in vitro

**Fig. 4:**
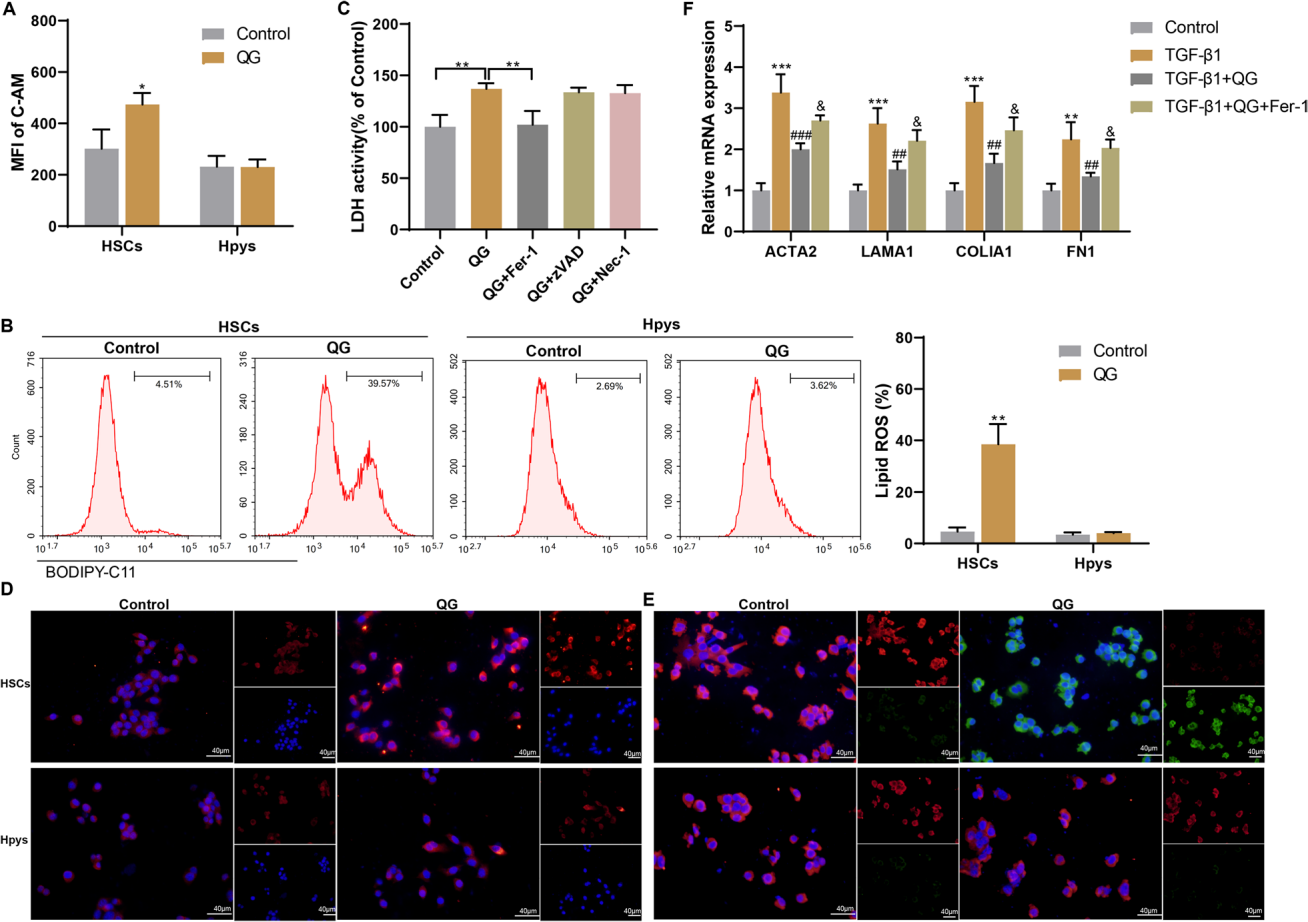
QG promotes ferroptosis of HSCs and inhibits HSC activation in vitro. **A, B** Primary mouse HSCs or Hpys were divided into control and QG groups. **A** MFI of intracellular C-AM was determined by flow cytometry. **B** Lipid ROS were detected by flow cytometry. **P < 0.01 *vs* control. **C** Primary mouse HSCs were divided into control, QG, QG + Fer-1, QG + zVAD, and QG + Nec-1 groups. LDH activity was detected. **P < 0.01 *vs* control or QG. **D–F** Mouse primary HSCs were divided into control, TGF-β1, TGF-β1 + QG, and TGF-β1 + QG + Fer-1 groups. The QG-induced ferroptosis was confirmed by utilizing FerroOrange staining (**D**) and BODIPY C11 staining (**E**). **F** The mRNA levels of ACTA2, LAMA1, COLlA1 and FN1 were detected by qRT-PCR. **P < 0.01, ***P < 0.001 *vs* control; ^##^*P* < 0.01, ^###^*P* < 0.001 *vs* TGF-β1; ^&^*P* < 0.05 *vs* TGF-β1 + QG

Metabonomic results showed that Fe^2+^ levels were increased in HFHCD mice after HGTLF administration (data not shown), suggesting that HGTLF and its active ingredients may ameliorate liver fibrosis by regulating ferroptosis. Since the discovery of ferroptosis as a new death pattern, more and more evidence has shown that ferroptosis plays a key role in the onset and development of liver diseases. Here, flow cytometry analysis showed an increase in intracellular LIP in primary mouse HSCs treated with QG, but had no significant effect on the intracellular LIP of Hpys (Fig. 4A). In addition, QG treatment increased lipid ROS in HSCs, but had no significant effect on lipid ROS in Hpys (Fig. 4B). Next, to verify that QG promotes ferroptosis in HSCs, primary mouse HSCs were treated with Fer-1 (ferroptosis inhibitor), zVAD (apoptosis inhibitor), or Nec-1 (necroptosis inhibitor). Figure 4C showed that Fer-1 inhibited QG-induced HSC death, whereas zVAD and Nec-1 had no significant effect on HSC death. The QG-induced ferroptosis was also confirmed by utilizing FerroOrange staining and BODIPY C11 staining. FerroOrange staining showed that levels of Fe^2+^ were increased in HSCs after QG treatment (Fig. 4D) and QG treatment increased lipid peroxidation levels in HSCs (Fig. 4E). Further in vitro experiments showed that Fer-1 treatment reversed the effect of QG on TGF-β1-induced HSCs, increasing ACTA2, LAMA1, COLlA1, and FN1 mRNA levels (Fig. 4F). Fer-1 has also been added for in vivo experiments. Due to the long induction period of the HFHCD mouse model, we utilized CCl_4_-injected mice to verify the effect of Fer-1 on the pharmacodynamic effect of QG. The results showed that Fer-1 partially attenuated the pharmacodynamic effect of QG in improving NAFLD (Fig. S3). The above results confirmed that Fer-1 treatment attenuated the inhibitory effect of QG on TGF-β1-induced HSC activation, indicating that QG promotes HSC ferroptosis and thus reduces HSC activation.

### QG promotes ubiquitination and degradation of GPX4

**Fig. 5:**
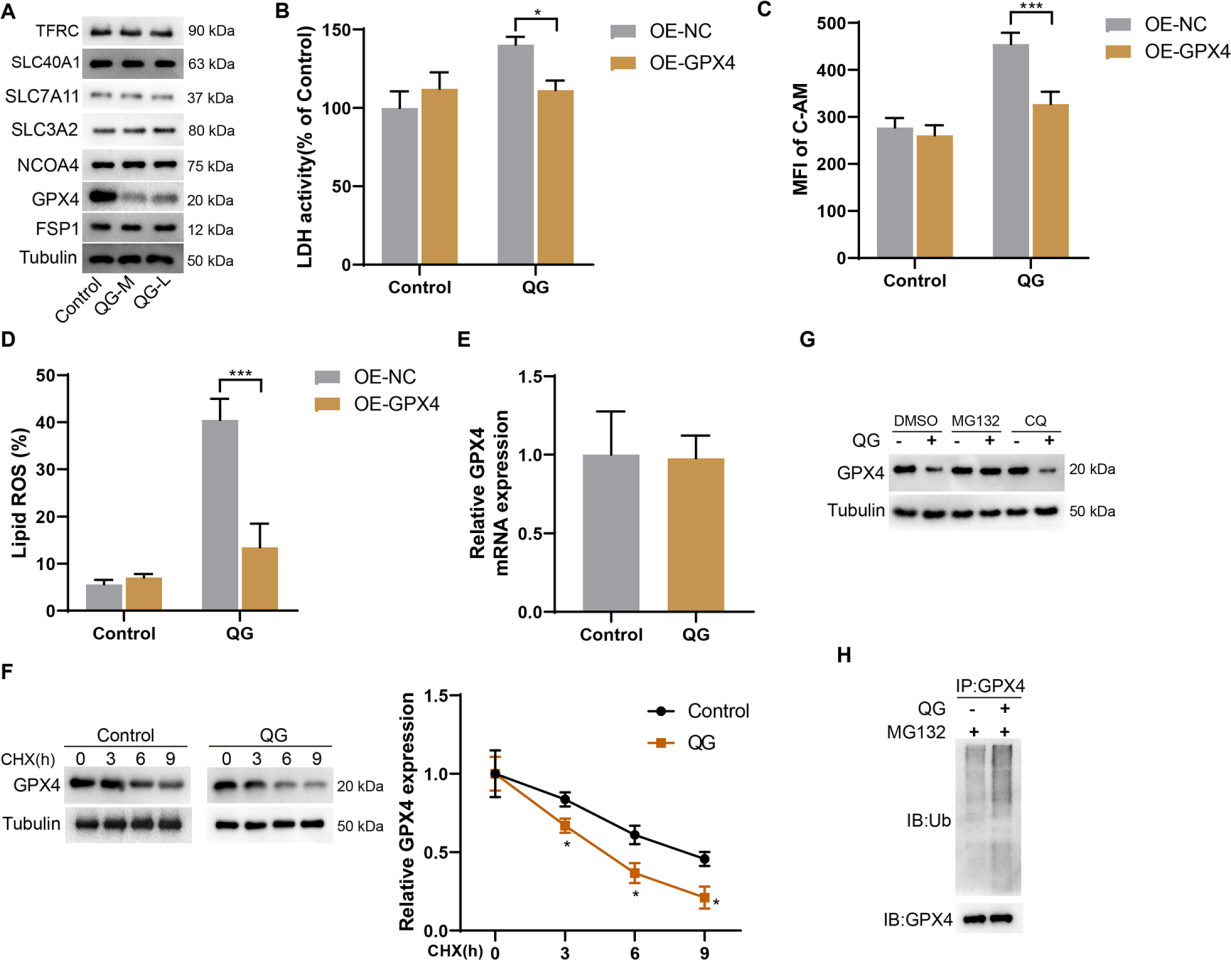
QG promotes ubiquitination and degradation of GPX4. **A** Mouse primary HSCs were divided into control, QG-M, and QG-L groups. Western blot was used to detect the protein expression of TFRC, SLC40A1, SLC7A11, SLC3A2, NCOA4, GPX4, and FSP1. **B–D** Primary HSCs were divided into control + OE-NC, control + OE-GPX4, QG + OE-NC, and QG + OE-GPX4 groups. **B** LDH activity was detected. **C** MFI of intracellular C-AM was determined by flow cytometry. **D** Lipid ROS was detected by flow cytometry. *P < 0.05, ***P < 0.001 *vs* QG + OE-NC. **E** The mRNA levels of GPX4 in the control and QG groups were detected by qRT-PCR. **F** CHX chase assay was used to assess the stability of GPX4 protein after the treatment of QG. *P < 0.05 *vs* control. **G** Western blotting of GPX4 protein expressions after the treatment of MG132 or CQ. **H** Ubiquitination of GPX4 protein in QG-treated primary mouse HSCs

The mechanism of ferroptosis is complex and there are many regulation mechanisms involving TFRC [11], NCOA4 [20], SLC40A1 [21], system Xc^−^ (SLC7A11 and SLC3A2) [22], GPX4 [23], FSP1 [24] and other proteins. First, Western blotting was used to detect the protein expressions of TFRC, SLC40A1, SLC7A11, SLC3A2, NCOA4, GPX4, and FSP1 in the control, QG-M, and QG-L groups. The results showed that QG inhibited GPX4 protein expression, but had no significant effect on the expressions of other proteins, suggesting that QG may promote ferroptosis of HSCs by regulating GPX4 expression (Fig. 5A). To investigate whether GPX4 affects ferroptosis of HSCs, primary HSCs were divided into control + OE-NC, control + OE-GPX4, QG + OE-NC, and QG + OE-GPX4 groups. Compared with the QG + OE-NC group, GPX4 overexpression could reduce the LDH activity, intracellular LIP and lipid ROS of HSCs in the QG + OE-GPX4 group (Fig. 5B–D). Next, qRT-PCR results showed that QG had no significant effect on GPX4 mRNA expression (Fig. 5E). The expression of GPX4 in both control group and QG group decreased gradually with the extension of CHX treatment time. QG treatment shortened the half-life of GPX4, indicating that it could reduce the stability of GPX4 protein (Fig. 5F). The results of proteasome inhibitor (MG132) and lysosome inhibitor (CQ) experiments showed that the addition of MG132 treatment saved QG-mediated GPX4 protein instability, but CQ had no such effect (Fig. 5G). More and more studies have confirmed that the ubiquitin–proteasome pathway regulates GPX4 protein degradation [25, 26]. Therefore, ubiquitination experiments were performed, and the results showed that QG treatment promoted the ubiquitination level of GPX4 protein in primary mouse HSCs (Fig. 5H). Based on the above results, this study identified GPX4, a key protein in the promotion of ferroptosis by QG in HSCs, and found a degradation pathway by which QG reduced GPX4 protein expression.

### QG promotes the interaction between GPX4 and E3 ubiquitin ligase MARCHF8

**Fig. 6:**
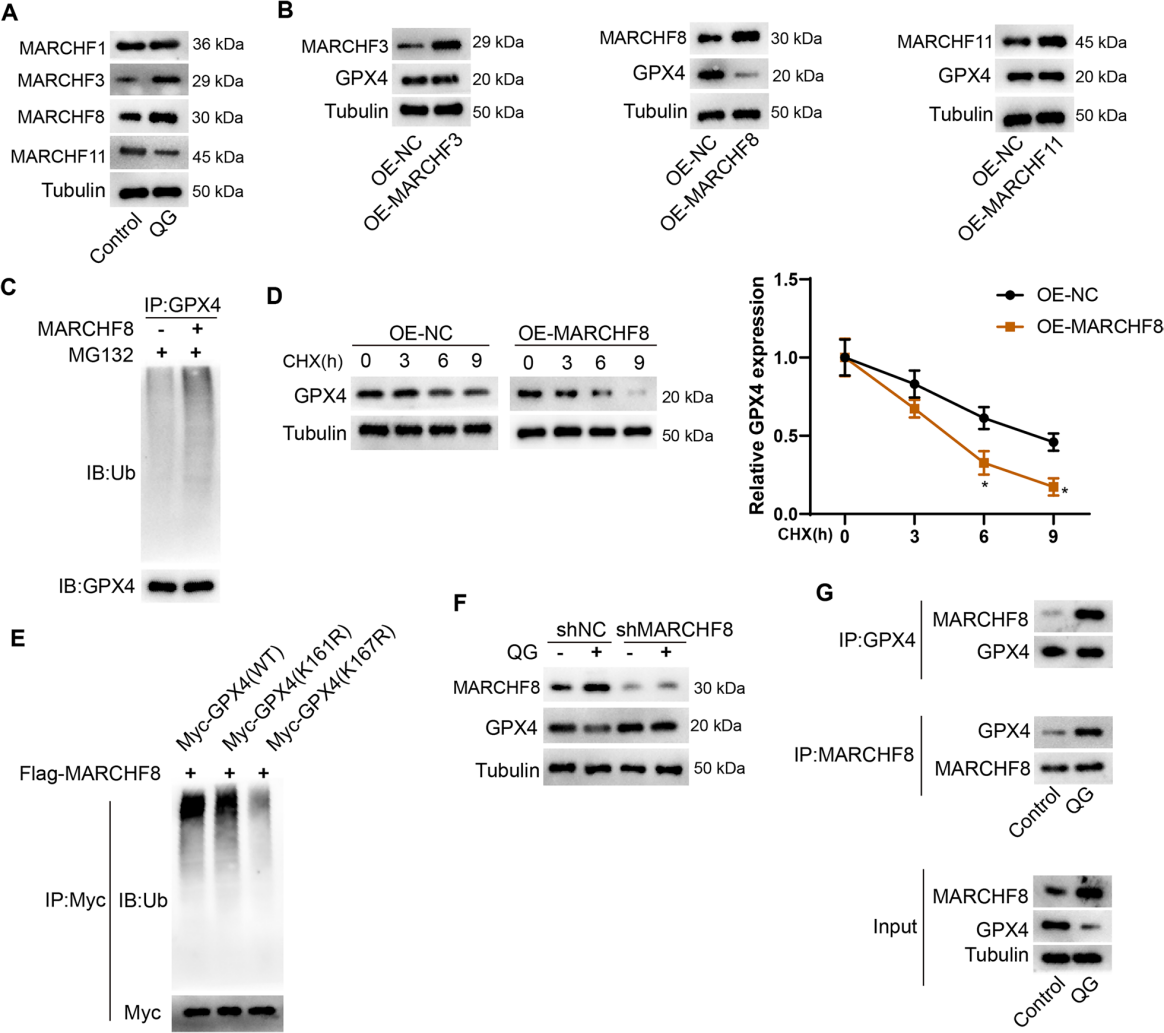
QG promotes the interaction between GPX4 and E3 ubiquitin ligase MARCHF8. **A** Mouse primary HSCs were divided into control and QG groups. Western blot of MARCHF1, MARCHF3, MARCHF8, MARCHF11 protein expression was performed. **B** Mouse primary HSCs were divided into OE-NC, OE-MARCHF1, OE-MARCHF3, OE-MARCHF8, OE-MARCHF11 groups. Western blotting for GPX4 protein expression was performed. **C** Ubiquitination of GPX4 protein in OE-MARCHF8 treated primary mouse HSCs. **D** CHX-chase assay was used to evaluate the stability of GPX4 protein after MARCHF8 overexpression treatment. *P < 0.05 *vs* OE-NC. **E** Ubiquitination of GPX4 protein was performed after K161 or K167 is mutated to R. **F** Primary mouse HSCs were divided into control + shNC, control + shMARCHF8, QG + shNC, and QG + shMARCHF8 groups. Western blot of MARCHF8 and GPX4 protein expressions was performed. **G** Co-IP experiment verified the interaction between GPX4 and MARCHF8

UbiBrowser 2.0 online database was used to predict the potential E3 ubiquitin ligases of GPX4 protein ubiquitin modification. The results showed that four E3 ubiquitin ligases, including MARCHF1, MARCHF3, MARCHF8, and MARCHF11, could regulate the ubiquitin modification of GPX4 protein. Therefore, we first examined the effect of QG on the expression of E3 ubiquitin ligases. As shown in Fig. 6A, after QG treatment, the protein expressions of MARCHF3 and MARCHF8 were increased, the protein expression of MARCHF11 was decreased, and MARCHF1 had no significant change. After overexpression of MARCHF3, MARCHF8 and MARCHF11 in primary mouse HSCs, it was found that OE-MARCHF8 decreased GPX4 protein expression, while overexpression of MARCHF3 and MARCHF11 had no significant effect on GPX4 protein expression (Fig. 6B). Further experiments showed that MARCHF8 overexpression promoted GPX4 ubiquitination and protein degradation (Fig. 6C, D). The UbiBrowser 2.0 online database predicted that MARCHF8 could mediate the ubiquitination of GPX4 K161 and K167. To investigate the specific site of GPX4 ubiquitination, 293 T cells were transfected with Flag-MARCHF8 plasmid, Myc-GPX4 (WT), Myc-GPX4 (K161R) or Myc-GPX4 (K167R). Figure 6E showed that the level of GPX4 ubiquitination was significantly reduced after the K167 mutation. These results indicated that MARCHF8 mediated GPX4 ubiquitination at K167. Next, primary mouse HSCs were transfected with MARCHF8 interfering plasmid or negative control and then treated with QG. Western blotting results showed that interfering with MARCHF8 effectively reversed the inhibitory effect of QG on GPX4 protein expression (Fig. 6F). We also found that the QG treatment group promoted the interaction between GPX4 and endogenous MARCHF8, and endogenous GPX4 could also be immunoprecipitated by MARCHF8, indicating the interaction between GPX4 and MARCHF8 (Fig. 6G). The above results indicated that the E3 ubiquitin ligase MARCHF8 mediated the ubiquitination of GPX4 K167, and QG promoted the ubiquitination and degradation of GPX4 by promoting the interaction between GPX4 and MARCHF8.

### QG promotes ferroptosis of HSCs and inhibits HSC activation through MARCHF8 mediated ubiquitination of GPX4

**Fig. 7:**
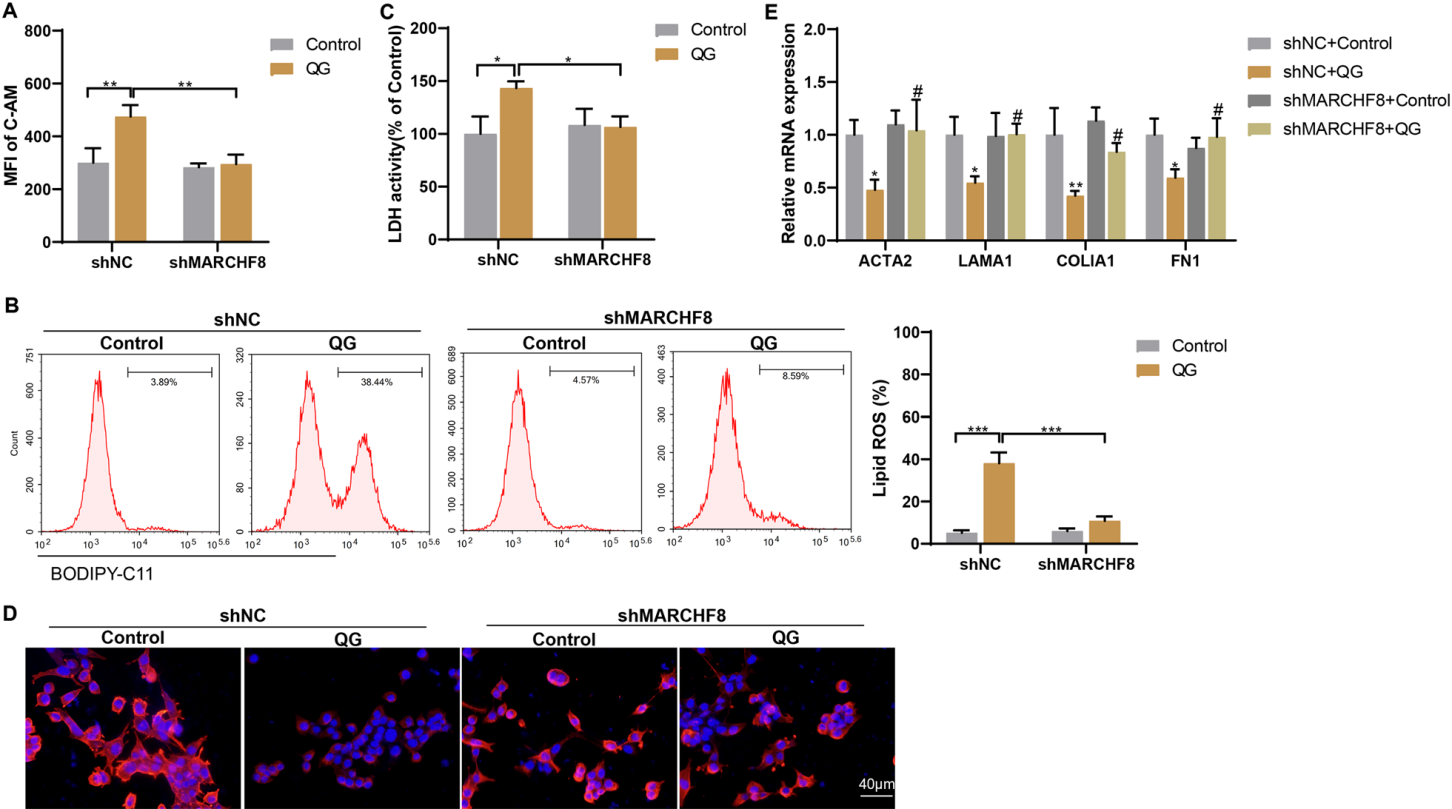
QG promotes ferroptosis of HSCs and inhibits HSCs activation through MARCHF8 mediated ubiquitination of GPX4. (A-C) Primary mouse HSCs were divided into control + shNC, control + shMARCHF8, QG + shNC, and QG + shMARCHF8 groups. **A** MFI of intracellular C-AM was determined by flow cytometry. **B** Lipid ROS was detected by flow cytometry. **C** LDH activity was detected. *P < 0.05, **P < 0.01, ***P < 0.001. **D–E** TGF-β1-treated primary mouse HSCs were divided into control + shNC, control + shMARCHF8, QG + shNC, and QG + shMARCHF8 groups. **D** Positive expression of α-SMA was detected by immunofluorescence staining (scale bar, 40 μm). **E** The mRNA levels of ACTA2, LAMA1, COLlA1, and FN1 were detected by qRT-PCR. *P < 0.05, **P < 0.01 *vs* shNC + Control. ^#^*P* < 0.05 *vs* shMARCHF8 + Control

Next, the effects of MARCHF8 interference on ferroptosis and activation of HSCs were examined. Compared with the OE + shNC group, MARCHF8 interference effectively reversed the QG-induced increase in LIP, lipid ROS, and HSC death in HSCs (Fig. 7A–C), indicating that QG promotes ferroptosis of HSCs by regulating MARCHF8. Furthermore, α-SMA immunofluorescence staining and fibrosis-related gene expression detection showed that interference with MARCHF8 effectively attenuated QG and promoted TGF-β1-induced HSC activation (Fig. 7D–E). The above in vitro experiments demonstrated that QG promoted HSC ferroptosis and inhibited HSC activation through MARCHF8-mediated GPX4 ubiquitination.

## Discussion

The mechanism of the onset and development of NAFLD is still unclear, and current therapeutic options are relatively limited. Therefore, more and more studies have begun to explore the potential targets and feasibility of NAFLD treatment. The development of anti-NAFLD drugs based on traditional Chinese medicine has the advantages and characteristics of syndrome differentiation, multi-component, multi-target and multi-pathway. Flavonoids have a variety of pharmacological activities and play an important role in improving lipid metabolism, anti-inflammation, antioxidation and regulation of abnormal cell apoptosis [27]. Recent studies have shown that flavonoid compounds of Chinese herbal medicine have significant anti-liver fibrosis effects [28]. Silybinin, a flavonoid used in clinical practice, can be used in the treatment of liver fibrosis, indicating that flavonoids have the potential to be developed into anti-liver fibrosis drugs [29]. QG, also known as hexahydroxyflavone, is a flavonoid component with great medicinal potential. It has been reported that QG can effectively reduce the risk of cancer and cardiovascular disease, and has the potential to be used as an antioxidant and anti-inflammatory agent due to its rich hydroxyl group [30]. In addition, QG also has α-glucosidase, α-amylase and pancreatic lipase inhibitory activities, which can be used as a potential therapeutic agent for diabetes and hyperlipidemia [31]. Therefore, the clinical development of QG is of great significance. In this study, a fibrosis mouse model was established by CCl_4_ injection and a NASH mouse model was established by HFHCD feeding to investigate the effects of QG gavage on liver lipid deposition and liver fibrosis in mice. AST, ALT and ALP are important indices to evaluate liver function, and TC and TG are important indices to evaluate lipid metabolism [32]. The above indexes were detected in vivo, and the results suggested that QG has a better effect on the lipid metabolism disorder caused by NAFLD. In addition, the pathological results of liver tissues showed that QG could reduce collagen deposition, inhibit collagen fiber formation, and delay the progression of liver fibrosis.

Liver fibrosis, characterized by the accumulation of ECM and collagen, is an important turning point in the development of NAFLD and has always been the focus and difficulty of clinical management of liver disease [33]. As chronic liver injury progresses to fibrosis, most liver cells (including parenchymal and non-parenchymal cells) release cytokines, including TGF-β1, which in turn activate HSCs to differentiate into myofibroblasts and secrete α-SMA and ECM proteins [34]. In vitro experiments confirmed that TGF-β1 treatment significantly promoted the expressions of α-SMA and fibrosis-related genes (ACTA2, LAMA1, COLlA1 and FN1) in HSCs. In addition, in vitro QG treatment promoted the death of primary HSCs and LX-2 cells. Ferroptosis is a new type of cell death caused by iron-dependent lipid peroxidation, which has been confirmed to play an important role in the pathological mechanism of liver fibrosis in the progression of various liver diseases, but its detailed mechanism needs to be further explored [35]. Zhang et al*.* found that treatment with ferroptosis inducers erastin and sorafenib can reduce liver fibrosis in bile duct ligation mice model by inducing iron deposition and thereby inducing ferroptosis in HSCs [36]. Therefore, inducing ferroptosis of activated HSCs is an effective way to inhibit the progression of liver fibrosis. To verify whether QG promoted ferroptosis of HSCs, rescue experiments were conducted using ferroptosis inhibitor Fer-1, which could inhibit QG-induced HSCs death. It is thought that ferroptosis is mainly mediated by exogenous (transporter-dependent) and endogenous (enzyme-regulated) pathways, where endogenous pathways are activated by blocking intracellular antioxidant enzymes, including GPX4 and FSP1 [24, 37]. System Xc^−^ composed of SLC7A11 and SLC3A2, participates in the synthesis of glutathione and GPX4 and inhibits ferroptosis by regulating the transport of glutamic acid and cysteine [38]. Recent studies have shown that traditional Chinese medicine berberine inhibits liver fibrosis by inducing ferroptosis in HSCs by inhibiting the SLC7A11/GPX4 axis [39]. Isoliquiritigenin can inhibit the expression of GPX4 and increase the expression of TFR and divalent metal ion transporter 1, produce a large amount of ROS, induce HSCs ferroptosis, and alleviate liver fibrosis [40]. Here, QG can inhibit GPX4 protein expression, and the overexpression of GPX4 partially eliminates the role of QG in promoting ferroptosis of HSCs. In conclusion, targeted induction of HSCs ferroptosis is a new promising target for the treatment of liver fibrosis, which is a potential treatment method.

Post-translational modification, including succination, ubiquitination, phosphorylation, and alkylation, is an important way to regulate GPX4 protein expression and function. Our study found that the ubiquitin proteasome-dependent pathway is involved in the downregulation of GPX4 protein induced by QG. MARCH family proteins are a recently discovered class of E3 ubiquitin ligases with an atypical RING-CH domain that can ubiquitinate target proteins, thereby regulating the stability, trafficking and function of various membrane proteins [41]. MARCHF8 belongs to one of the 11 members of the MARCH family and was first identified as a cellular regulator of immune recognition [42]. Further experiments showed that MARCHF8 mediated the ubiquitination of GPX4 at K167 site, and MARCHF8 can interact with GPX4. In addition, in vitro experiments found that interference with MARCHF8 effectively attenuated the effect of QG and promoted TGF-β1-induced HSCs activation.

During the degradation of GPX4 protein, specific modification types of ubiquitination sites (such as monoubiquitination, polyubiquitination, and chain linking mode) are involved. Previous studies have shown that the polyubiquitination modifications of GPX4 protein are mainly K48-linked polyubiquitination and K63-linked polyubiquitination. Among them, K48-linked polyubiquitination usually marks GPX4 protein, which is recognized and degraded by proteasome, leading to the decrease of GPX4 protein level, weakening its antioxidant capacity and promoting lipid peroxidation and ferroptosis [43]. K63-linked polyubiquitination does not directly lead to the degradation of GPX4 protein, but may affect its signaling and protein interactions [44]. K63-linked polyubiquitination of GPX4 has been poorly studied, and its role in ferroptosis needs to be further investigated. Therefore, after identifying that QG regulates GPX4 proteasome-mediated degradation, our pre-experiment detected and confirmed that QG promotes polyubiquitination modification of K48-linked polyubiquitination of GPX4 protein using a K48-specific ubiquitin antibody. Based on these studies, we speculate that different types of ubiquitination modifications may regulate the biological functions of GPX4 by affecting stability, activity, and localization, which we plan to further explore in follow-up studies.

This study was based on animal model and cell experiments to observe the molecular mechanism of QG inhibition of HSC activation, but there are still shortcomings, that is, lack of clinical trials. We will investigate this in future studies.

**Fig. 8:**
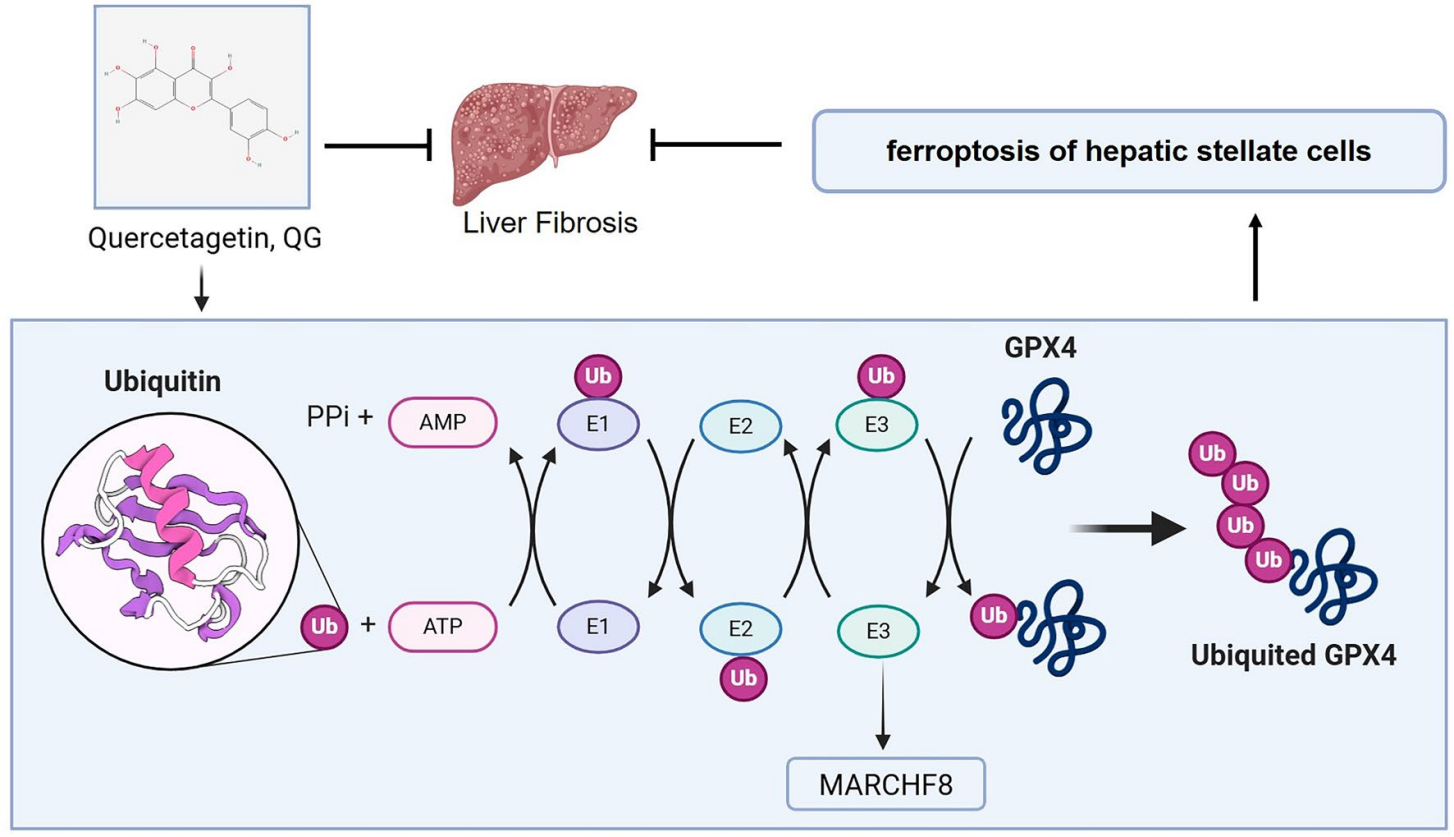
QG can effectively inhibit HSC activation through targeted induction of HSC ferroptosis, thereby alleviating lipid deposition and liver fibrosis. QG promotes HSC ferroptosis and inhibits HSC activation through MARCHF8-mediated ubiquitination of GPX4

In conclusion, QG can effectively inhibit HSCs activation through targeted induction of HSCs ferroptosis, thereby alleviating lipid deposition and liver fibrosis in CCl_4_ injection and NASH model mice, suggesting that QG may be one of the key agents of HGTLF against liver fibrosis (Fig. 8). This study tentatively revealed the mechanism of HGTLF compound against liver fibrosis in NAFLD and provided scientific data to support its clinical application.

## Supplementary Information

Supplementary Materials 1: Fig.S1.Screening of key active ingredients in HGTLF. (A) The composition of HGTLF was determined by UPLC-MS/MSin the cation and anion ionization modes. Mice were divided into HFHCD group and HFHCD+HGTLF group (N=3/group). (B-D) Effective components absorbed into blood from HGTLF were operated by using UPLC-MS/MS in the cation and anion ionization modesand analyzed. (E) HPLC was used to quantitatively detect all flavonoids in mouse liver tissues (N=5).

Supplementary Materials 2: Fig.S2. QG alleviates liver lipid deposition by inhibitingHSC activation. (A) Mice were divided into NCD, NCD+QG, HFHCD, HFHCD+QG groups (N=5/group). The expressions of p-AMPK, AMPK, SREBP-1c, and PPAR-γ were detected using Western blotting. (B) Mice were divided into control, FFA, FFA+QG groups (N=5/group). Theexpressions of p-AMPK, AMPK, SREBP-1c, and PPAR-γ were detected using Western blotting.

Supplementary Materials 3: Fig.S3. QG improves NAFLD by promoting ferroptosis.(A) Mice were divided into control (corn oil), CCl4, CCl4+QG, CCl4+QG+Fer-1 groups (N=5/group). (B) Blood samples of mice in each group were collected for detection of AST, ALT and ALP levels. (C) The liver tissues of mice in each group were collected for detection of TC and TG contents. (D) The liver tissues of mice in each group were stained with HE (scale bar, 50 μm), Masson (scale bar, 50 μm) and Sirius red (scale bar, 100 μm). (E) HYO levels were detected in the liver tissues of mice in each group to characterize the content of collagen. *P < 0.05, **P < 0.01, ***P < 0.001.

Supplementary Materials 4.

## Data Availability

The datasets used and/or analyzed during the current study are available from the corresponding author on reasonable request.

## Abbreviations

NAFLD: Nonalcoholic fatty liver disease
NASH: nonalcoholic steatohepatitis
HGTLF: Huagan tongluo Fang
HSCs: Hepatic stellate cells
α-SMA: α-smooth muscle actin
ECM: extracellular matrix
GPX4: glu-tathione peroxidase 4
UPLC-MS/MS: ultra-high performance liquid chromatography tandem mass spectrometry
QG: Quercetagetin
TGF-β1: transforming growth factor-β1
HFHCD: high-fat and high-cholesterol diet
NCD: normal chow diet
TG: triglyceride
TC: total cholesterol
AST: aspartate aminotransferase
ALT: alanine aminotransferase
ALP: alkaline phosphatase
HE: Hematoxylin-eosin
HYP: hydroxyproline
DAB: 3, 3 N-diaminobenzidine tertrahydrochloride
Hpys: hepatocytes
Fer-1: Ferrostatin-1
zVAD: zVAD-FMK
Nec-1: Necrostatin-1
ACTA2: α-2 smooth muscle actin
LAMA1: laminin 1
COLlA1: collagen type1 α-1
FN1: fibronectin 1
LDH: Lactate dehydrogenase
C-AM: calcien acetoxymethyl ester
MFI: mean fluorescence intensity
TFRC: transferrin receptor
SLC40A1: solute carrier family 40 member 1
SLC7A11: solute carrier family 7 member 11
SLC3A2: solute carrier family 3 member 2
NCOA4: nuclear receptor co-activator 4
GPX4: glutathione peroxidase 4
FSP1: ferroptosis-suppressor-protein 1
MARCHF1: membrane-associated ring-CH-type finger 1
CHX: Cycloheximide
CQ: chloroquine
HEPES: N-(2-hydroxyethyl)piperazine-N'-2-ethanesulfonic acid
Co-IP: Co-immunoprecipitation
