# Supplementary figures and images for: Quercetagetin alleviates liver fibrosis in non-alcoholic fatty liver disease by promoting ferroptosis of hepatic stellate cells through GPX4 ubiquitination
Source: Chin Med. 2025 Jun 16;20:89. doi: 10.1186/s13020-025-01109-x (PMC12168282; doi:10.1186/s13020-025-01109-x)

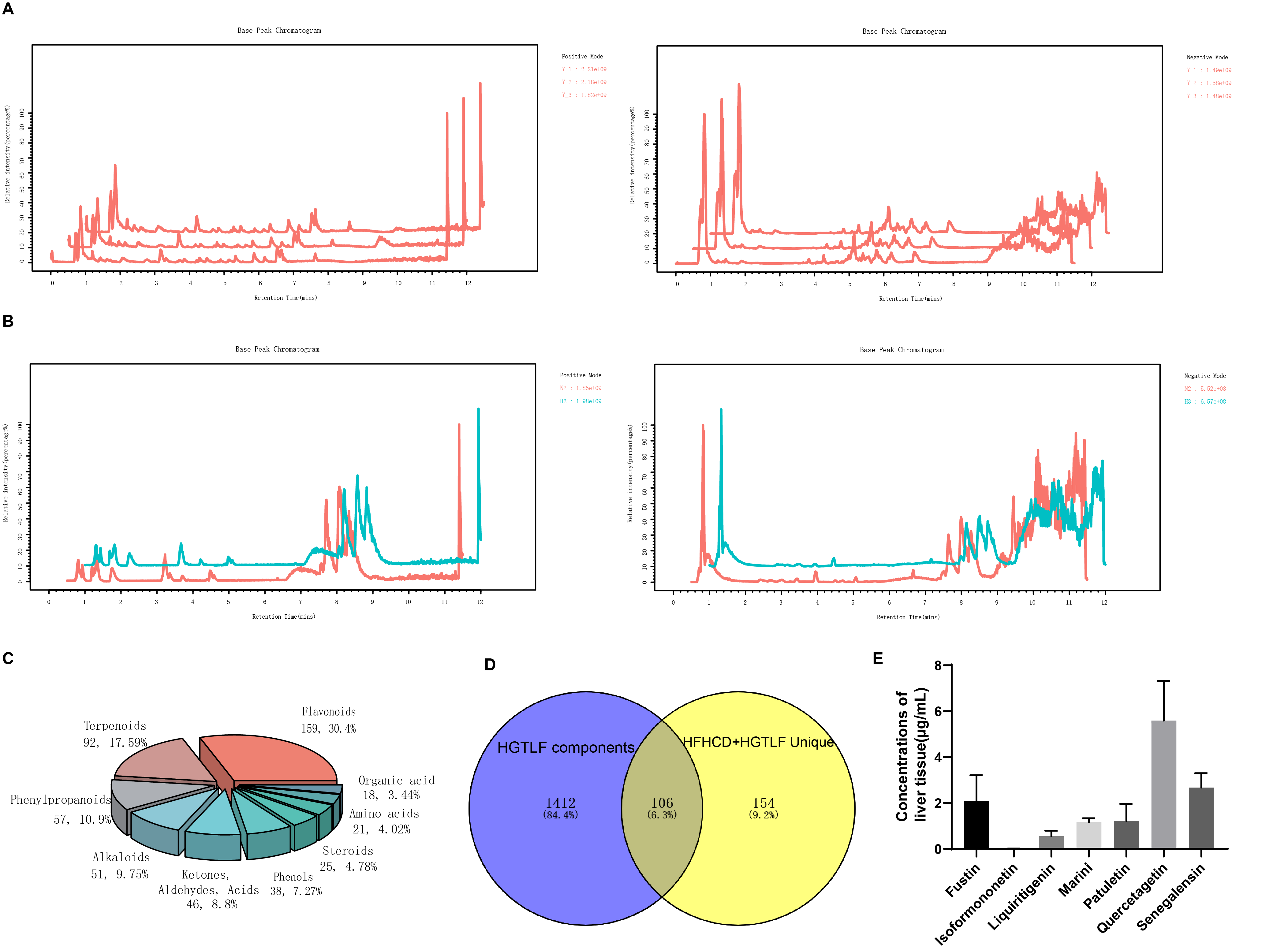

Supplement: Supplementary file 1 — Supplementary Materials 1: Fig.S1.Screening of key active ingredients in HGTLF. (A) The composition of HGTLF was determined by UPLC-MS/MSin the cation and anion ionization modes. Mice were divided into HFHCD group and HFHCD+HGTLF group (N=3/group). (B-D) Effective components absorbed into blood from HGTLF were operated by using UPLC-MS/MS in the cation and anion ionization modesand analyzed. (E) HPLC was used to quantitatively detect all flavonoids in mouse liver tissues (N=5). [file 13020_2025_1109_MOESM1_ESM.tif]

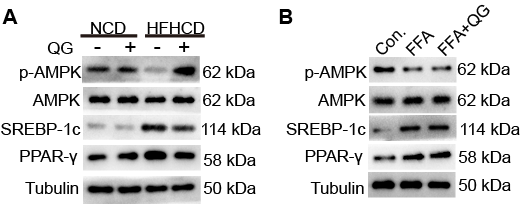

Supplement: Supplementary file 2 — Supplementary Materials 2: Fig.S2. QG alleviates liver lipid deposition by inhibitingHSC activation. (A) Mice were divided into NCD, NCD+QG, HFHCD, HFHCD+QG groups (N=5/group). The expressions of p-AMPK, AMPK, SREBP-1c, and PPAR-γ were detected using Western blotting. (B) Mice were divided into control, FFA, FFA+QG groups (N=5/group). Theexpressions of p-AMPK, AMPK, SREBP-1c, and PPAR-γ were detected using Western blotting. [file 13020_2025_1109_MOESM2_ESM.tif]

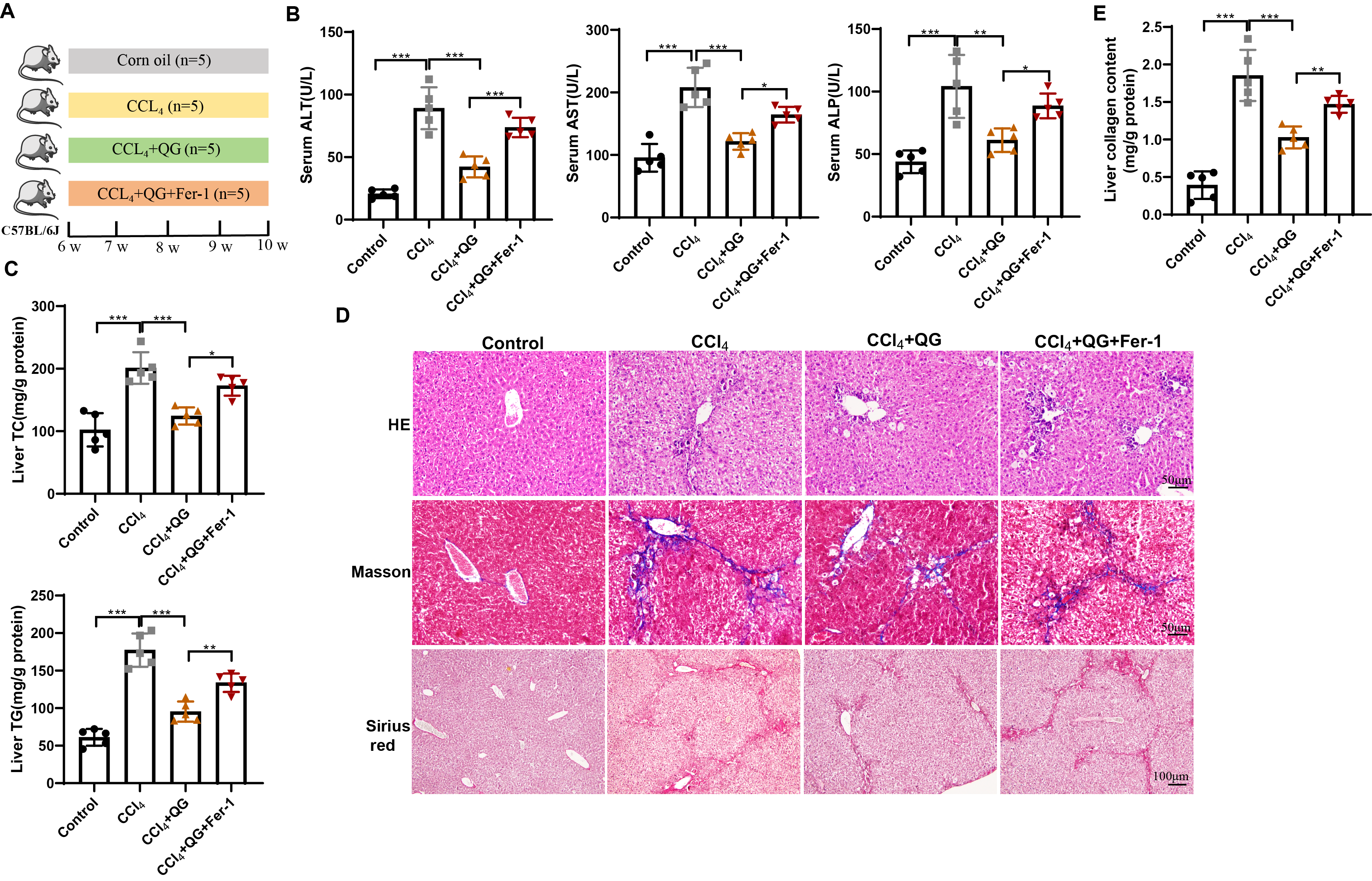

Supplement: Supplementary file 3 — Supplementary Materials 3: Fig.S3. QG improves NAFLD by promoting ferroptosis.(A) Mice were divided into control (corn oil), CCl4, CCl4+QG, CCl4+QG+Fer-1 groups (N=5/group). (B) Blood samples of mice in each group were collected for detection of AST, ALT and ALP levels. (C) The liver tissues of mice in each group were collected for detection of TC and TG contents. (D) The liver tissues of mice in each group were stained with HE (scale bar, 50 μm), Masson (scale bar, 50 μm) and Sirius red (scale bar, 100 μm). (E) HYO levels were detected in the liver tissues of mice in each group to characterize the content of collagen. *P < 0.05, **P < 0.01, ***P < 0.001. [file 13020_2025_1109_MOESM3_ESM.tif]
